# A role for 3′ exonucleases at the final stages of chromosome duplication in *Escherichia coli*

**DOI:** 10.1093/nar/gky1253

**Published:** 2018-12-14

**Authors:** Sarah L Midgley-Smith, Juachi U Dimude, Christian J Rudolph

**Affiliations:** Division of Biosciences, College of Health and Life Sciences, Brunel University London, Uxbridge UB8 3PH, UK

## Abstract

Chromosome duplication initiates via the assembly of replication fork complexes at defined origins, from where they proceed in opposite directions until they fuse with a converging fork. Recent work highlights that the completion of DNA replication is highly complex in both pro- and eukaryotic cells. In this study we have investigated how 3′ and 5′ exonucleases contribute towards the successful termination of chromosome duplication in *Escherichia coli*. We show that the absence of 3′ exonucleases can trigger levels of over-replication in the termination area robust enough to allow successful chromosome duplication in the absence of *oriC* firing. Over-replication is completely abolished if replication fork complexes are prevented from fusing by chromosome linearization. Our data strongly support the idea that 3′ flaps are generated as replication fork complexes fuse. In the absence of 3′ exonucleases, such as ExoI, these 3′ flaps can be converted into 5′ flaps, which are degraded by 5′ exonucleases, such as ExoVII and RecJ. Our data support the idea that multiple protein activities are required to process fork fusion intermediates. They highlight the complexity of fork fusions and further support the idea that the termination area evolved to contain fork fusion-mediated pathologies.

## INTRODUCTION

Every time a cell divides, its entire DNA content has to be replicated with high fidelity and the copies accurately transmitted to the daughter cells (1). Any failure to do so can be lethal for cells, or lead to mutation and genomic instability, the root causes of cancer. As is the case with many other bacteria, *Escherichia coli* carries a single circular chromosome. Replication is initiated at the single origin of replication, *oriC*, via the action of the main initiator protein DnaA, which facilitates assembly of two replication fork complexes called replisomes. Once established, both replisomes move away from *oriC* in opposite directions, replicating the DNA with very high speed and accuracy (2). Replication concludes when both replisomes fuse opposite *oriC* within the termination region of the chromosome (3,4). In *E. coli*, this area is flanked by polar *ter* sequences (*terA–J*), binding sites for the Tus terminator protein. *ter*/Tus complexes are polar and can pause a fork approaching from one direction, but are permissive for forks moving in the opposite direction. The *ter* sites form clusters in such a way that the termination area forms a replication fork trap which replication forks can enter but not leave (3–5). This divides the chromosome into two approximately equal ‘replichores,’ each replicated by a single replisome (6). In addition, the terminus region contains specialised genetic elements such as the *dif* site, which facilitates the resolution of chromosome dimers, and KOPS sequences, which guide proteins facilitating the segregation of duplicated DNA to daughter cells (7,8).

In *E. coli*, initiation and elongation of DNA replication, but also many of the final steps of chromosome duplication, such as decatenation and chromosome dimer resolution, have been extensively studied and are generally well understood (2,7,9). In contrast, the events associated with the fusion of two replisomes have only recently received significant attention. It has become apparent that a surprisingly large number of proteins are involved in the processing of intermediates that accumulate specifically in the area where replication forks fuse, including RecG helicase, the nucleases ExoI, ExoVII and SbcCD, DNA polymerase I and RecBCD recombinase/exonuclease (3,10–18). Recent studies suggest that RecG helicase is one of the key players dealing with intermediates specifically arising as replication forks fuse (10,13,16,19). RecG is a multifunctional DNA translocase that is capable of remodelling a variety of branched DNA structures *in vitro* (20–22). Marker frequency analyses (MFA) of exponentially growing cells lacking RecG revealed substantial levels of over-replication in areas where replication forks fuse, both in the native but also in an ectopic fork fusion area (10,13,16,17,19). These observations suggest that events associated with termination of DNA replication have the potential to trigger aberrant DNA synthesis, and that RecG normally curbs such events. The fact that over-replication can be triggered in an ectopic location rules out the involvement of any sequence element specific to the termination area (13).

**Figure 1.**
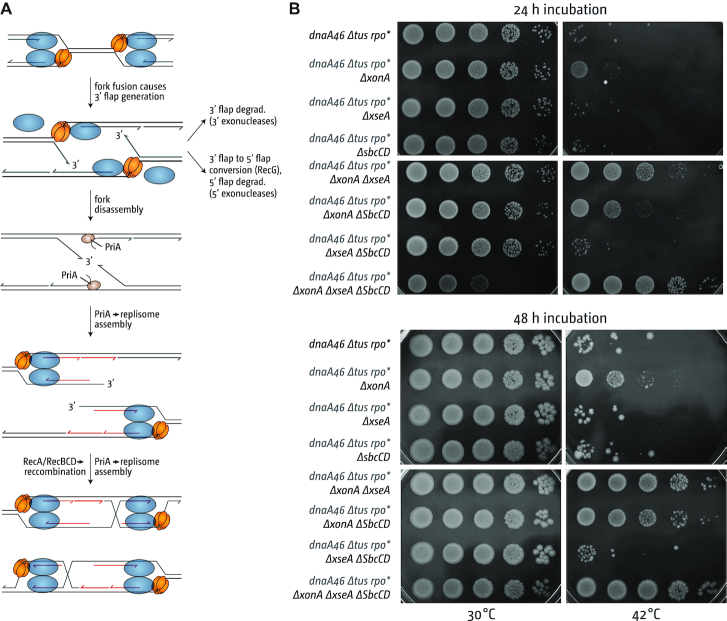
Replication initiated at fork fusion intermediates can result in DNA replication which can sustain cell growth. (**A**) Schematic illustrating how replication fork fusions might lead to the formation of new divergent forks via PriA-mediated replisome assembly and RecBCD-mediated recombination and how this can be normally suppressed by RecG and 3′ exonucleases. See text for details. (**B**) DnaA-independent growth triggered by the absence of 3′ exonucleases. Spot dilution assay showing the effect of *xonA* (ExoI), *xseA* (ExoVII) and *sbcCD* (SbcCD) mutations on growth without DnaA (*dnaA46* at 42°C). The replication fork trap in the termination area was inactivated by deletion of *tus* and an rpoB*35 point mutation was introduced to alleviate replication-transcription conflicts resulting from replication forks travelling in a direction opposite to normal. The strains used were RCe267 (dnaA46 Δtus rpo*), RCe528 (dnaA46 Δtus rpo* ΔxonA), SLM1219 (dnaA46 Δtus rpo* ΔxseA), RCe553 (dnaA46 Δtus rpo* ΔsbcCD), SLM1194 (dnaA46 Δtus rpo* ΔxonA ΔxseA), RCe554 (dnaA46 Δtus rpo* ΔxonA ΔsbcCD), SLM1223 (dnaA46 Δtus rpo* ΔxseA ΔsbcCD) and SLM1226 (dnaA46 Δtus rpo* ΔxonA ΔxseA ΔsbcCD).

We have suggested previously that during the fusion of two replication forks the DnaB helicase of one fork might sometimes displace the leading strand of the opposing fork, resulting in the formation of a 3′ ssDNA flap structure (Figure 1A) (10,11,16). Such flaps would normally be rapidly eliminated. 3′ single-stranded flaps indeed are an excellent substrate for RecG helicase, which possesses the necessary activity to unwind the 5′ end at the branch point of a 3′ flap while simultaneously reannealing the 3′ single-strand flap (20,23–25). Thus, RecG could convert a 3′ flap structure into a 5′ flap, which then would be removed by a 5′ single-stranded DNA exonuclease. Alternatively, 3′ flaps could be directly removed by 3′ exonucleases. In the absence of either RecG or 3′ exonucleases, such 3′ flaps are likely to persist for longer and can be processed by the main replication fork restart protein PriA, which will trigger the assembly of a new replication fork (Figure 1A). Progression of such a fork will result in the generation of a double-stranded DNA end. Any such DNA end will be rapidly processed by homologous recombination proteins RecBCD, which will load the RecA recombinase, thereby triggering the invasion of the re-replicated DNA behind the fork (Figure 1A) or the sister duplex (not shown) (3,10,14–16,22). Such an invasion would establish a D-loop, another substrate for PriA, where another replication fork could be established, thereby restoring bi-directional DNA synthesis which would proceed in a direction opposite to normal (Figure 1A). This model is able to explain that (a) over-replication in cells lacking RecG is significantly reduced if the chromosome is linearized near *dif*, which prevents the fusion of forks in the termination area (10,16), (b) over-replication in cells lacking RecG depends on RecA and RecBCD recombinases, but not the RuvABC Holliday junction resolvase (13,16) and (c) over-replication in cells lacking RecG requires the helicase activity of PriA and, more specifically, its ability to process 3′ flaps (10,16).

The model predicts that cells lacking the ability to remove 3′ ssDNA flaps should show over-replication of the termination area. In line with this idea, it was observed that in cells lacking the exonucleases ExoI and SbcCD (17), but in particular in cells lacking all three major 3′ exonucleases ExoI, ExoVII and SbcCD, significant levels of over-replication are observed in the termination area (16). ExoI, ExoVII and SbcCD are all nucleases involved in aspects of DNA repair. However, their precise cellular function remains poorly understood. ExoI, encoded by the *xonA* gene, is a processive 3′ to 5′ exonuclease that can degrade up to 10 000 nucleotides per minute (26). It was shown that ExoI binds to the C-terminal domain of SSB (27,28), together with other proteins such as RecJ exonuclease, RecG helicase and other repair and genome maintenance proteins (29). ExoVII consists of two subunits that are encoded by *xseA* and *xseB. xseA* encodes the catalytically active subunit, while *xseB* encodes for a smaller subunit which is likely to regulate the activity of ExoVII (26). In contrast to Exo I, ExoVII is capable of processively degrading both 3′ and 5′ ends (30, 31). Both ExoI and ExoVII are involved in methyl-directed mismatch repair in *E. coli* (32,33). Finally, the dimeric SbcCD nuclease has both a 3′ exonuclease activity and endonuclease activity that can cut secondary structures formed by inverted repeats or palindromic sequences, which results in the formation of a double-strand break (26,34). It can remove 3′ ssDNA overhangs from double-strand breaks, thereby making the end accessible for the loading of RecBCD, which requires blunt or near-blunt dsDNA ends (26).

In this study, we further define the roles 3′ exonucleases play in preventing over-replication in the terminus area of the chromosome. We show that the DNA synthesis triggered in the termination area of cells lacking 3′ exonucleases is robust enough to allow successful chromosome duplication in the absence of *oriC* firing, despite the fact that levels of over-replication observed by MFA are relatively low. Growth in the absence of *oriC* firing strictly requires the inactivation of the replication fork trap. In addition, 3′ exonuclease-deficient cells are no longer able to grow in the absence of oriC firing if the chromosome is linearized, highlighting that the over-replication initiated in the termination area is key for driving successful chromosome duplication in the absence of origin activity. Our data strongly support the idea that 3′ exonucleases have an important role in processing intermediates that arise as a consequence of replication fork fusion events in the termination area and their defined substrate specificity strongly supports the idea that 3′ flaps are a key fork fusion intermediate. The data presented suggest that other proteins such as RecG might be able to process at least some of the accumulating intermediates, highlighting a certain degree of overlap in the pathways known to be involved in the processing of termination intermediates. However, our data also indicate that there are some differences in the intermediates processed by RecG and by 3′ exonucleases.

## MATERIALS AND METHODS

### Bacterial strains and general methods

For *E. coli* K12 strains see Supplementary Table S1. Strains were constructed via P1*vir* transductions (35) or by single-step gene disruptions (36). The *dnaA46* allele encodes a thermosensitive DnaA protein that is inactive at 42°C. For assessing growth without DnaA initiation, cultures of *dnaA46* constructs grown at 30°C to an *A*_600_ of 0.4 were diluted in 10-fold steps from 10^–1^ to 10^–5^ before spotting 10 μl samples of each dilution on LB agar. Duplicate plates were incubated at 30°C and 42°C.

### Growth media

Luria broth (LB) and agar was modified from Luria and Burrous (37) as follows: 1% tryptone (Bacto™, BD Biosciences), 0.5% yeast extract (Bacto™, BD Biosciences) and 0.05% NaCl (Sigma Aldrich). The pH was adjusted to 7.4. Mu broth for bacteriophage P1 and N15 work contained 1% tryptone (Bacto™, BD Biosciences), 0.5% yeast extract (Bacto™, BD Biosciences) and 1% NaCl (Sigma Aldrich). The pH was adjusted to 7.4.

### Synthetic lethality assay

The synthetic lethality assay was performed as described (38,39). In essence, a wild type copy of a gene of interest (*xonA*) under its native promoter was cloned into pRC7, a *lac^+^* mini-F plasmid that is rapidly lost, and used to cover the deletion of the same gene in the chromosome in a *Δlac^–^* background (see Supplementary Information for plasmid details). Additional mutations can then be introduced to test for synthetic lethality with the deleted allele. If synthetically lethal, cells that lose the plasmid will fail to grow and only *lac^+^* colonies formed by cells retaining the plasmid will be observed. When viability is reduced but not eliminated, colonies formed by cells retaining the plasmid are noticeably larger than white colonies formed by plasmid-free cells. Cultures of strains carrying the relevant pRC7 derivatives were grown overnight in LB broth containing ampicillin to maintain plasmid selection, diluted 100-fold in LB broth and grown without ampicillin selection to an *A*_600_ of 0.4 before spreading dilutions on LB agar or M9 glucose minimal salts agar supplemented with X-gal and IPTG. Plates were photographed and scored after 48 h (LB agar) or 72 h (M9 agar) at 37°C.

### Marker frequency analysis by deep sequencing

Marker frequency analysis by deep sequencing was performed as described before (40). In brief, fresh overnight cultures were diluted 100-fold in fresh LB broth and incubated with vigorous aeration until an *A*_600_ reached 0.48 at 37°C. The cultures were then diluted again 100-fold in pre-warmed fresh broth and grown again until an *A*_600_ of 0.48 was reached. Samples from these exponential phase cultures were flash-frozen in liquid nitrogen for subsequent DNA extraction. For a wild type stationary phase sample, incubation of the remaining culture was continued until several hours after the culture had saturated and showed no further increase in the *A*_600_. A further sample was then frozen. DNA was then extracted using the GenElute Bacterial Genomic DNA Kit (Sigma-Aldrich). Marker frequency analysis was performed using Illumina HiSeq 2500 sequencing (fast run) to measure sequence copy number. The enrichment of uniquely mapped sequence tags in 1 kb windows was then calculated. Replication profiles of all key constructs were confirmed by two independent experiments. See the Supplementary Methods section for a more detailed description.

### Linearization of the *E. coli* chromosome

Linearization of the *E. coli* chromosome was performed as described before (16,41). In brief, the *tos* linearization site of bacteriophage N15 was integrated near the *dif* chromosome dimer resolution site into the *E. coli* chromosome. Cells were then infected with N15 and lysogenic cells collected. Lysogenic infection with N15 leads to expression of the N15 telomerase, TelN, which processes the *tos* linearization site, thereby causing linearization of the *E. coli* chromosome. Successful linearization was confirmed by PCR and pulsed-field gel electrophoresis. See Supplementary Methods and Supplementary Figure S1 for further details.

## RESULTS

### Growth of cells lacking 3′ exonucleases in the absence of origin firing

Initiation of chromosome replication is normally strictly regulated by DnaA, the main initiator protein in *E. coli* (42). However, marker frequency analysis (MFA) of exponentially growing cells revealed a substantial level of over-replicated sequences within the termination region in cells in which RecG helicase is missing (10,16). This over-replication in *ΔrecG* cells is sufficiently strong to maintain chromosome replication in the absence of *oriC* firing and cells lacking RecG can tolerate deletion of the entire *oriC* area, but only if fork traps are eliminated by deletion of *tus* and conflicts between replication and transcription reduced by an rpo* mutation, (10,16), a mutation that destabilises RNA polymerase transcription complexes, thereby alleviating replication-transcription conflicts (40,43–45).

Given that cells lacking 3′ exonucleases also show substantial levels of over-replication in the termination area (16,17), we wanted to investigate whether 3′ exonuclease-deficient cells can grow in the absence of *oriC* firing. We used a dnaA(ts) Δtus rpo* background to generate derivatives with single deletions of *xonA, xseA* and *sbcCD*, all combinations of double deletions and a strain lacking all three genes. All constructs grew well overall at 30°C (Figure 1B; see Supplementary Table S2 for an extended analysis of doubling times, viability and DNA damage sensitivity of all dnaA(ts) Δtus rpo* constructs used in this study). dnaA(ts) Δtus rpo* cells lacking all three 3′ exonuclease genes showed a mild slow-growth phenotype as indicated by the smaller colony sizes following incubation for 24 h (Figure 1B), but incubation for 48 h confirmed that the level of viability is not significantly affected in these cells.

At restrictive temperature the contribution of the 3′ exonuclease genes in terms of growth shows a clear hierarchy. Only dnaA(ts) Δtus rpo* ΔxonA cells show some growth at 42°C, whereas for both dnaA(ts) Δtus rpo* ΔxseA and dnaA(ts) Δtus rpo* ΔsbcCD growth does not appear to be different in comparison to the dnaA(ts) Δtus rpo* control. However, if *ΔxonA* is combined with either *ΔxseA* or *ΔsbcCD*, the ability to grow is substantially increased. dnaA(ts) Δtus rpo* ΔxonA ΔxseA cells grow robustly at 42°C, and just over 60% of cells growing at 30°C were also able to form colonies at 42°C (61 ± 16.5%), a very similar level to that observed in dnaA(ts) Δtus rpo* ΔrecG cells (16). dnaA(ts) Δtus rpo* ΔxonA ΔsbcCD cells grow robustly, but the ability to grow is mildly reduced. Thus, absence of ExoI clearly has the strongest effect, followed by ExoVII and then SbcCD. A combination of *xseA* and *sbcCD* deletions resulted in only a very minor increase in the ability of dnaA(ts) Δtus rpo* cells to grow at 42°C. Nevertheless, a deletion of *sbcCD* clearly increased the ability of dnaA(ts) Δtus rpo* ΔxonA ΔxseA cells to grow at 42°C (Figure 1B), demonstrating that all three 3′ exonucleases do contribute to the ability to grow in the absence of origin firing.

**Figure 2.**
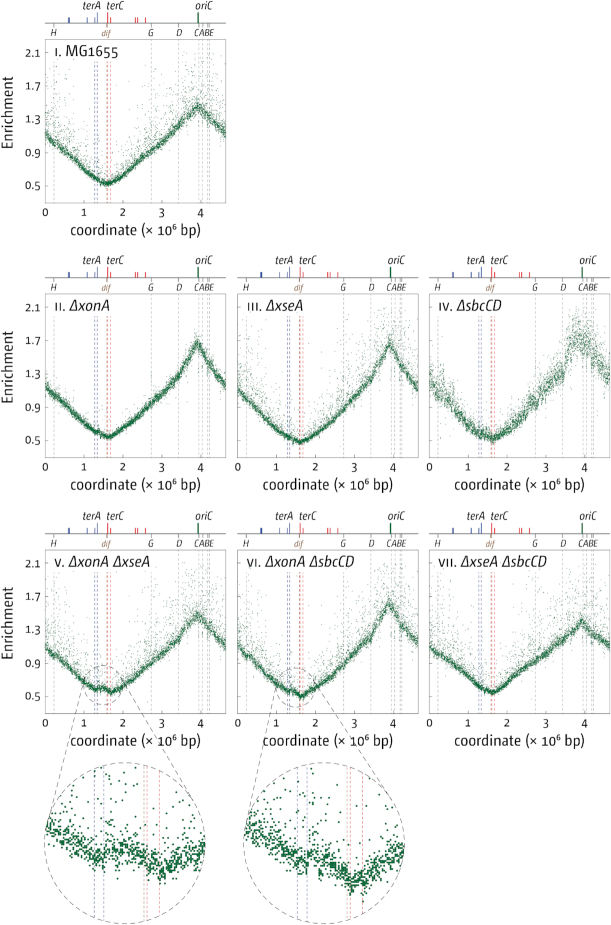
Over-replication in the termination area of *E. coli* cells in the presence and absence of 3′ exonuclease proteins. The number of reads (normalised against reads for a stationary phase wild type control) is plotted against the chromosomal location. A schematic representation of the *E. coli* chromosome showing positions of *oriC* and *ter* sites (above) as well as *dif* and *rrn* operons *A–E, G* and *H* (below) is shown above the plotted data. Sequencing templates were isolated from MG1655 (wild type), RCe563 (*ΔxonA*), SLM1185 (*ΔxseA*), RCe562 (*ΔsbcCD*), SLM1203 (*ΔxonA ΔxseA*), RCe569 (*ΔxonA ΔsbcCD*) and SLM1209 (*ΔxseA ΔsbcCD*).

We were surprised to find that despite the clear growth shown in Figure 1B, over-replication in the termination area of 3′ exonuclease single and double mutants is much more subtle than the over-replication observed in cells lacking RecG (10,13,16), suggesting that levels of over-replication do not directly correlate with the ability to grow in the absence of origin firing. The difference in the level of over-replication also suggests that some differences in the molecular mechanisms of how the over-replication is triggered in cells lacking either RecG of 3′ exonucleases exist. None of the single mutants showed any significant increase of the marker frequency in the termination area (Figure 2), but some over-replication in the termination area is seen in both *ΔxonA ΔxseA* and *ΔxonA ΔsbcCD* double mutants.

The strongest level of growth (Figure 1) and over-replication (16) was observed in cells lacking all three 3′ exonucleases. However, dnaA(ts) Δtus rpo* ΔxonA ΔxseA ΔsbcCD cells clearly show a growth defect (Figure 1), and *ΔxonA ΔxseA ΔsbcCD* triple mutants are sensitive to DNA damage, whereas *ΔxonA ΔxseA* are not (22) (Supplementary Table S2). For this reason we decided to focus our studies on dnaA(ts) Δtus rpo* ΔxonA ΔxseA cells, as growth in these cells is robust enough at 42°C for all genetic tests.

**Figure 3.**
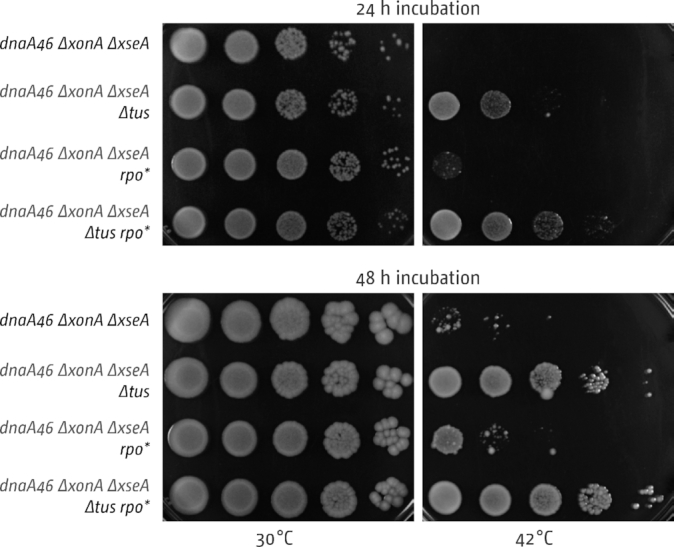
Effect of Tus and RNA polymerase mutations on growth and DNA synthesis of *dnaA46* strains lacking 3′ exonucleases ExoI (*ΔxonA*) and ExoVII (*ΔxseA*). Spot dilution assay showing the effect of *Δtus* and rpoB*35 mutations on the ability of *dnaA(ts) ΔxonA ΔxseA* cells to grow at restrictive temperature. The strains used were SLM1246 (*dnaA46 ΔxonA ΔxseA*), SLM1244 (*dnaA46 ΔxonA ΔxseA Δtus*), SLM1245 (dnaA46 ΔxonA ΔxseA rpo*) and SLM1194 (dnaA46 ΔxonA ΔxseA Δtus rpo*).

The relatively low level of over-replication observed in the replication profile of cells lacking 3′ exonuclease might suggest relatively high levels of initiation events that might be evenly distributed throughout the chromosome. Indeed it was shown that recombination-dependent replication in *Haloferax volcanii* strains in which all origins have been deleted is so evenly distributed on a population basis that no distinct peaks are observable (46). To verify that growth of dnaA(ts) Δtus rpo* ΔxonA ΔxseA cells at 42°C is maintained by the over-replication in the termination area and not by replication initiated elsewhere in the chromosome, we investigated the dependency of growth on the deletion of *tus*, to allow DNA synthesis to escape the termination area, as well as an rpo* point mutation, which reduces replication-transcription conflicts (40,43–45). As shown in Figure 3, *dnaA(ts) ΔxonA ΔxseA* cells were unable to grow at 42°C. An rpo* point mutation increased growth marginally, while the deletion of *tus* had a much more significant effect. A combination of both mutations had the strongest effect, as observed before in cells lacking RecG (16). These results strongly suggest that growth in the absence of origin firing is mostly maintained by replication initiated in the termination area, similar to the situation reported in cells lacking RecG (10,16). As the termination area spans almost 45% of the *E. coli* chromosome we cannot exclude that initiation events might take place away from the fork fusion area. However, we reported before that in cells lacking ExoI, ExoVII and SbcCD a rather dramatic peak of over-replication is observed within the innermost *ter* sites, with little indication of replication taking place anywhere else. Even minor initiation sites were detectable via replication profiles in cells lacking RNase HI (10,47,48). Thus, if synthesis is initiated elsewhere within the broad termination area, levels of synthesis must be such that they do not lead to any visible distortion of the replication profile.

### Over-replication of the termination area in cells lacking 3′ exonucleases is abolished if the chromosome is linearized

**Figure 4.**
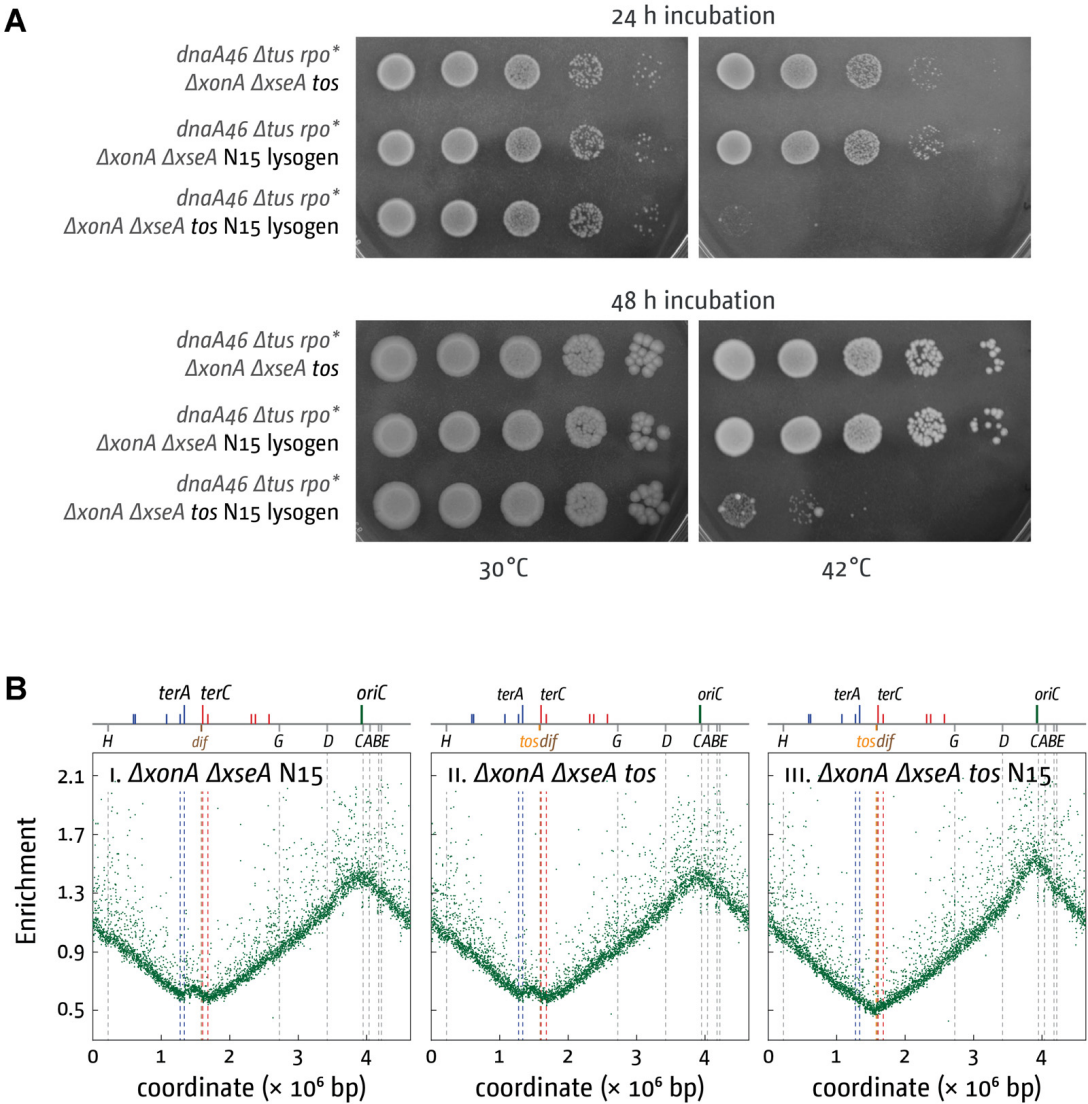
Effect of chromosome linearisation on DnaA-independent growth and replication of strains lacking 3′ exonucleases ExoI (*ΔxonA*) and ExoVII (*ΔxseA*). (**A**) Spot dilution assay showing the effect of chromosome linearization on the ability of dnaA(ts) Δtus rpo* ΔxonA ΔxseA cells to grow at restrictive temperature. The strains used were SLM1225 (dnaA46 Δtus rpo* ΔxonA ΔxseA tos), SLM1232 (dnaA46 Δtus rpo* ΔxonA ΔxseA N15 lysogen) and SLM1230 (dnaA46 Δtus rpo* ΔxonA ΔxseA tos N15 lysogen). (**B**) Marker frequency analysis of *E. coli ΔxonA ΔxseA* cells with a linearized chromosome. The number of reads (normalised against reads for a stationary phase wild type control) is plotted against the chromosomal location. A schematic representation of the *E. coli* chromosome showing positions of *oriC* and *ter* sites (above) as well as *dif* and *rrn* operons *A–E, G* and *H* (below) is shown above the plotted data. Sequencing templates were isolated from SLM1213 (*ΔxonA ΔxseA* N15 lysogen), SLM1187 (*ΔxonA ΔxseA tos*) and SLM1212 (*ΔxonA ΔxseA tos* N15 lysogen).

We demonstrated before that over-replication in *ΔrecG* cells is significantly reduced if the chromosome is linearized at the *dif* chromosome dimer resolution site in the termination area (10,16). In cells with a linearized chromosome replication forks coming from *oriC* will never meet and merge, but will instead run into chromosome ends (Supplementary Figure S1). Thus, the reduction of over-replication in *ΔrecG* cells with a linearized chromosome strongly supports the idea that fork fusion events cause the formation of intermediates that can trigger the observed over-replication (10,16). To investigate whether the same was true for cells lacking 3′ exonucleases, we introduced the linearization site *tos* near to the *dif* chromosome dimer resolution site into dnaA(ts) Δtus rpo* ΔxonA ΔxseA cells. Linearization was then achieved via the subsequent lysogenic infection with bacteriophage N15 to allow generation of the N15 telomerase, TelN (41). As shown in Figure 4A, linearization of the chromosome essentially abolished growth of dnaA(ts) Δtus rpo* ΔxonA ΔxseA cells at 42°C. Integration of the *tos* linearization site in the absence of TelN, and lysogenic infection with N15 without the presence of the *tos* linearization site, had no noticeable effect. Replication profiles are in line with these results and confirmed that over-replication in the termination area of *ΔxonA ΔxseA* cells is unaffected by the integration of *tos* or the lysogenic infection with bacteriophage N15, while the combination of both effectively abolishes the over-replication observed (Figure 4B). Thus, both the growth of dnaA(ts) Δtus rpo*ΔxonA ΔxseA cells and the over-replication in the termination area of *ΔxonA ΔxseA* cells is abolished if the chromosome is linearized. The persistence of origin-independent growth and over-replication in the termination area in cells lacking 3′ exonucleases, but which carry the integrated *tos* linearization sequence, rules out that integration of *tos* into a cryptic origin might be responsible for the effect seen in cells with a linearized chromosome.

### Over-replication of the termination area in cells lacking 3′ exonucleases requires PriA helicase activity

**Figure 5.**
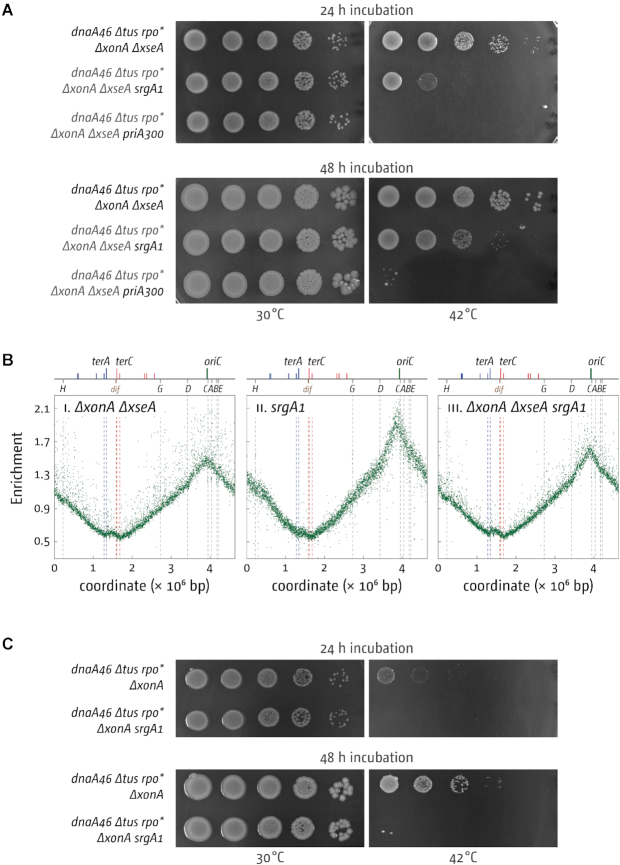
Effect of PriA helicase mutations on DnaA-independent growth and replication of strains lacking 3′ exonucleases ExoI (*ΔxonA*) and ExoVII (*ΔxseA*). (**A**) Spot dilution assay showing the effect of PriA helicase mutations on the ability of dnaA(ts) Δtus rpo* ΔxonA ΔxseA cells to grow at restrictive temperature. The strains used were SLM1194 (dnaA46 Δtus rpo*ΔxonA ΔxseA), SLM1199 (dnaA46 Δtus rpo* ΔxonA ΔxseA srgA1) and SLM1198 (dnaA46 Δtus rpo* ΔxonA ΔxseA priA300). (**B**) Marker frequency analysis of *E. coli ΔxonA ΔxseA* cells with PriA helicase mutations. The number of reads (normalised against reads for a stationary phase wild type control) is plotted against the chromosomal location. A schematic representation of the *E. coli* chromosome showing positions of *oriC* and *ter* sites (above) as well as *dif* and *rrn* operons *A–E, G* and *H* (below) is shown above the plotted data. Sequencing templates were isolated from SLM1203 (*ΔxonA ΔxseA*), JJ1264 (*srgA1*) and SLM1186 (*ΔxonA ΔxseA srgA1*). (**C**) Spot dilution assay showing the effect of *srgA1* on the ability of dnaA(ts) Δtus rpo* ΔxonA cells to grow at restrictive temperature. The strains used were SLM1152 (dnaA46 Δtus rpo*ΔxonA) and SLM1110 (dnaA46 Δtus rpo* ΔxonA srgA1).

We demonstrated before that the over-replication of the termination area in cells lacking RecG helicase is completely abolished if the helicase activity of the main replication restart protein PriA is inactivated (10,16). To investigate whether the same is the case in cells lacking 3′ exonucleases we constructed dnaA(ts) Δtus rpo* ΔxonA ΔxseA cells containing a *priA300* point mutation, which encodes the helicase-deficient PriA K230R (49). This almost entirely abolished the ability of dnaA(ts) Δtus rpo* ΔxonA ΔxseA cells to grow at 42°C, while growth at 30°C remained unaffected (Figure 5A), similar to our observations in cells lacking RecG (10,16).

Even more specifically, we found that the over-replication in cells lacking RecG is almost entirely abolished by the introduction of a *srgA1* point mutation. *srgA1* is an allele of *priA* that encodes a mutant protein (PriA L557P) with a very specific alteration of its biochemical substrate specificity. It unwinds a replication fork with both a leading and a lagging strand at the branch point as efficiently as wild type PriA, but it has lost the ability to unwind a fork in which the leading strand is missing (50). This substrate is the equivalent of a 3′ flap. We speculated that 3′ ssDNA flaps would accumulate in the absence of 3′ exonucleases, similar to the situation in cells lacking RecG. If true then the *srgA1* allele should also abolish over-replication in the termination area in 3′ exonuclease-deficient cells. However, this was not what we observed (Figure 5A). Introduction of a *srgA1* point mutation reduced growth of dnaA(ts) Δtus rpo* ΔxonA ΔxseA cells at 42°C about 10-fold. However, growth is by no means completely abolished and robust colony formation is seen in the less diluted samples, in line with our replication profiles which showed that the peak of over-replication in the termination area is still present in *ΔxonA ΔxseA srgA1* cells (Figure 5B). This result is much in contrast to cells lacking RecG where the reduction of growth caused by *srgA1* is rather substantial (10,16).

### Accumulating 5′ ssDNA flaps can contribute towards over-replication in the termination area

Why would over-replication in the termination area in cells lacking 3′ exonucleases not require the ability of PriA helicase to process 3′ flaps? Cells lacking one or multiple 3′ exonucleases still contain functional RecG protein, which could convert 3′ ssDNA flaps into 5′ flaps. In fact, in cells lacking all three 3′ exonucleases, ExoI, ExoVII and SbcCD, RecG becomes essential for viability (22). If 3′ flaps are converted into 5′ flaps, they would normally be degraded by 5′ exonucleases, such as ExoVII and RecJ, both of which can degrade ssDNA with a 5′ to 3′ polarity (26). PriA has the ability to restart replication at a 5′ flap structure (25,51). Thus, a prediction would be that the deletion of genes encoding for 5′ exonucleases, such as *xseA* or *recJ*, should result in an increase of over-replication in the termination area. To test this we first wanted to investigate whether the growth of dnaA(ts) Δtus rpo* ΔxonA ΔxseA srgA1 observed at 42°C was dependent on the *ΔxseA* deletion. Indeed, we observed that growth of dnaA(ts) Δtus rpo* ΔxonA cells at 42°C is completely abolished by a *srgA1* point mutation (Figure 5C). Thus, one explanation for the growth observed in dnaA(ts) Δtus rpo* ΔxonA ΔxseA srgA1 cells is that 3′ flaps accumulated in the absence of ExoI are converted into 5′ flaps and degraded by ExoVII and RecJ. In the absence of ExoVII these start to accumulate, giving PriA the opportunity to directly establish replication forks at 5′ flap substrates (25,51).

**Figure 6.**
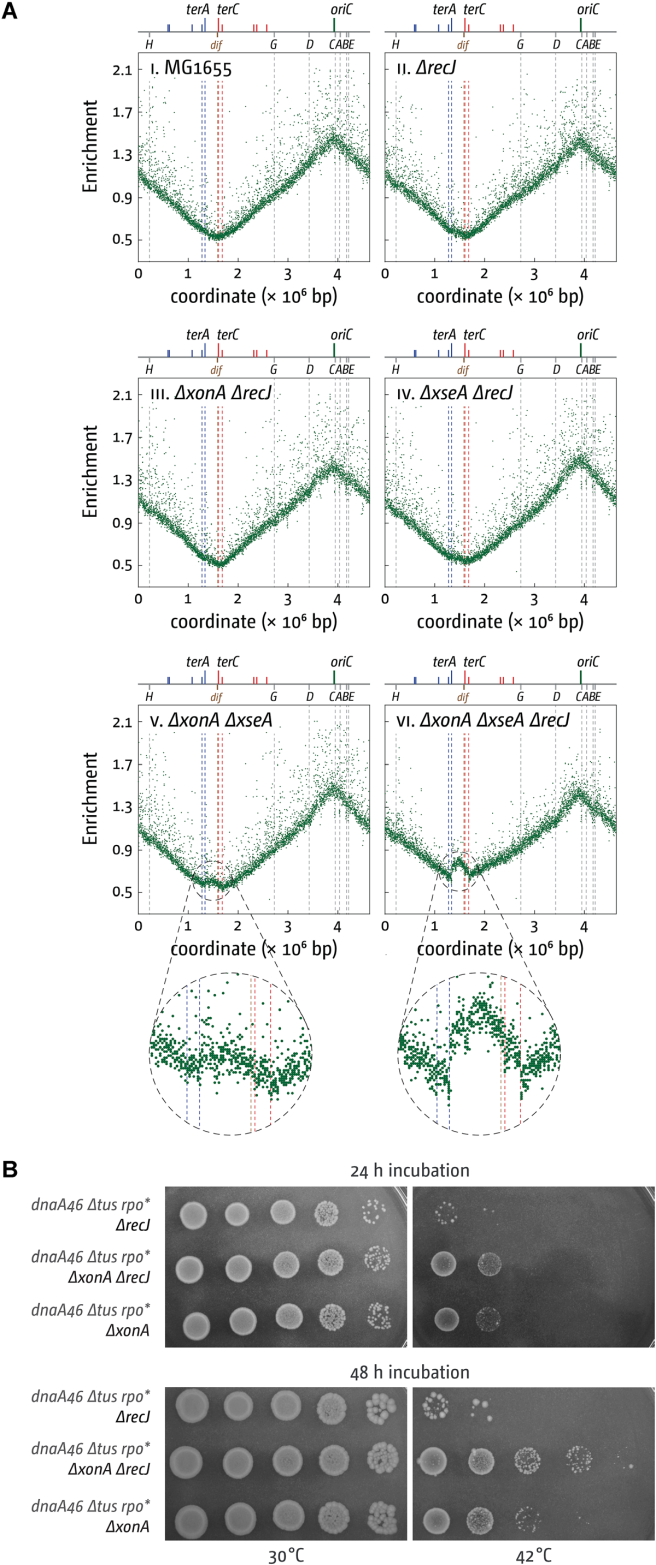
Effect of a *recJ* mutation on DnaA-independent growth and replication of strains lacking 3′ exonucleases ExoI (*ΔxonA*) and ExoVII (*ΔxseA*). (**A**) Marker frequency analysis of *E. coli ΔxonA, ΔxseA* and *ΔxonA ΔxseA* cells with a *recJ* mutation. The number of reads (normalised against reads for a stationary phase wild type control) is plotted against the chromosomal location. A schematic representation of the *E. coli* chromosome showing positions of *oriC* and *ter* sites (above) as well as *dif* and *rrn* operons *A–E, G* and *H* (below) is shown above the plotted data. Sequencing templates were isolated from MG1655 (wild type), N4934 (*ΔrecJ*), SLM1178 (*ΔrecJ ΔxonA*), SLM1204 (*ΔrecJ ΔxseA*), SLM1203 (*ΔxonA ΔxseA*) and SLM1188 (*ΔxonA ΔxseA ΔrecJ*). (**B**) Spot dilution assay showing the effect of a *recJ* deletion on the ability of dnaA(ts) Δtus rpo* ΔxonA cells to grow at restrictive temperature. The strains used were SLM1218 (dnaA46 Δtus rpo* ΔxonA), SLM1233 (dnaA46 Δtus rpo*ΔrecJ) and SLM1224 (dnaA46 Δtus rpo* ΔxonA ΔrecJ).

If this was the case then the deletion of *recJ* should also increase the ability of dnaA(ts) Δtus rpo* cells lacking 3′ exonucleases to grow at 42°C. Given that growth of dnaA(ts) Δtus rpo*ΔxonA ΔxseA cells is already very robust at 42°C, thereby obscuring any effect of a *recJ* deletion, we used dnaA(ts) Δtus rpo* cells with only the *xonA* gene deleted. In line with our prediction we observed that dnaA(ts) Δtus rpo* ΔxonA ΔrecJ cells showed improved growth at 42°C (Figure 6B). The effect is only moderate, but in these cells ExoVII is still present, which will interfere with the ability to grow at 42°C via its ability to degrade both 3′ and 5′ ends in a processive way (26). In line with the growth experiments, we found that the deletion of *recJ* caused a noticeable increase in the over-replication observed in the termination area of *ΔxonA ΔxseA* double mutants (Figure 6A).

### An asymmetric replichore arrangement does not increase over-replication of the termination area in cells lacking 3′ exonucleases

Over-replication in cells lacking RecG is dramatically increased if chromosome replication becomes asymmetric (13,16). In *ΔrecG* cells in which the chromosome is replicated from both the native *oriC* as well as an ectopic origin called *oriZ*, a copy of a 5 kb *oriC* fragment integrated roughly half way into the right-hand replichore, the peak of over-replication in the termination area even exceeds peak height of the native *oriC* (16). In this background replication coming from the ectopic *oriZ* will reach the termination area much earlier than forks coming from *oriC*, and forks are therefore blocked by *ter*/Tus complexes for some time (13,40,52). *In vitro* and *in vivo* measurements of replisome stability following arrest at different obstacles such as supercoiling and repressor-operator complexes suggest that active replisomes might have a half-life of 4–6 min (53–55). The extended period of arrest at *ter*/Tus complexes might therefore increase the likelihood of fork disassembly and subsequent processing by recombination enzymes, and we have suggested that a collision event between a moving replisome and such a disassembled or even processed fork will increase the chance of over-replication (13). In fact, *ΔoriC oriZ^+^ ΔrecG* cells proved inviable unless *tus* was deleted, demonstrating that the lethality of *ΔoriC oriZ^+^ ΔrecG* cells is caused by serious problems in the termination area (13).

**Figure 7.**
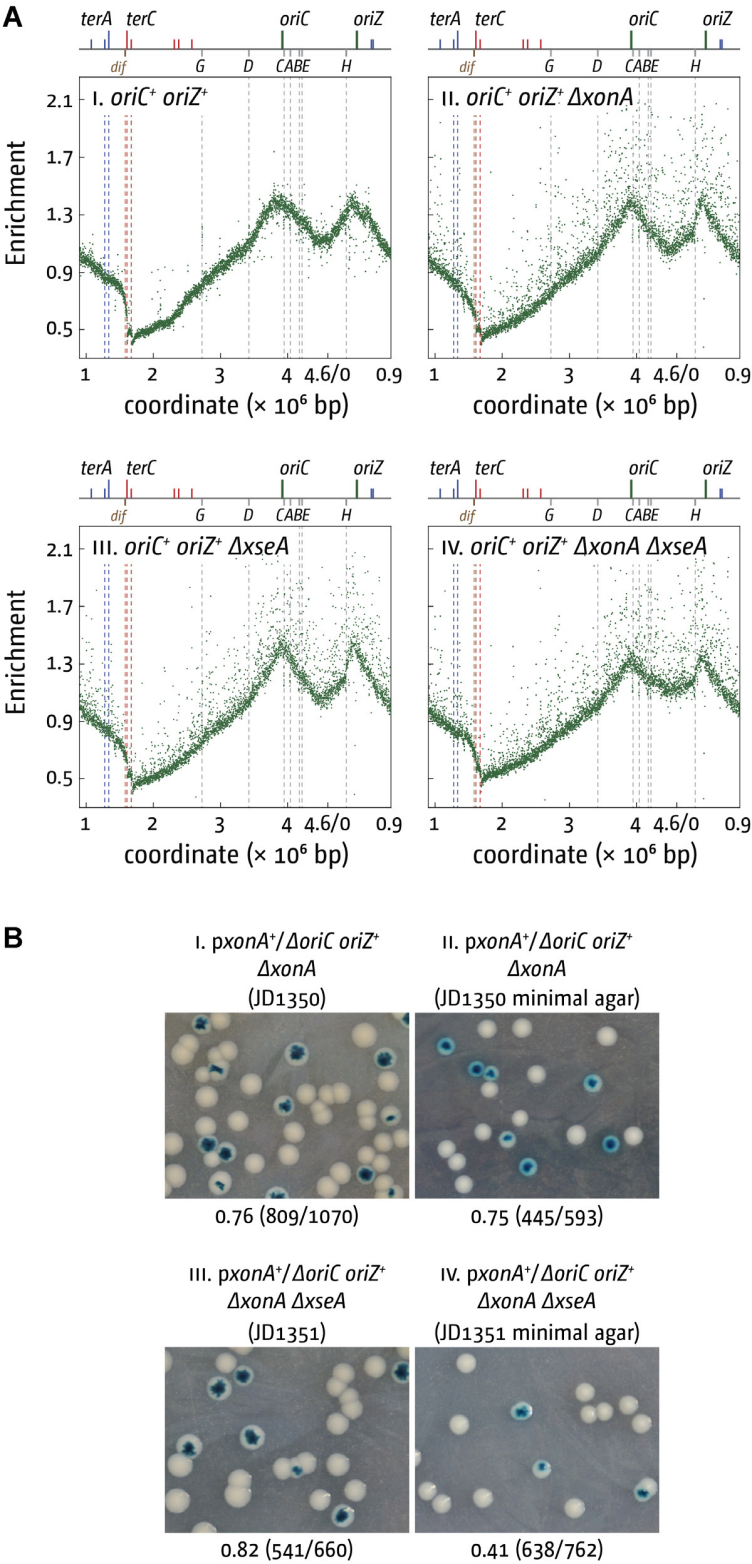
DNA replication and viability of *E. coli* cells with two replication origins in the absence of 3′ exonucleases ExoI (*ΔxonA*) and ExoVII (*ΔxseA*). **A**) Marker frequency analysis of *oriC^+^ oriZ^+^* cells lacking either ExoI, ExoVII or both ExoI and ExoVII. The number of reads (normalised against reads for a stationary phase wild type control) is plotted against the chromosomal location. A schematic representation of the *E. coli* chromosome showing positions of *oriC, oriZ* and *ter* sites (above) as well as *dif* and *rrn* operons *A–E, G* and *H* (below) is shown above the plotted data. Sequencing templates were isolated from RCe504 (*oriC^+^ oriZ^+^*), SLM1206 (*oriC^+^ oriZ^+^ ΔxonA*), SLM1208 (*oriC^+^ oriZ^+^ Δ*xseA) and SLM1217 (*oriC^+^ oriZ^+^ Δ*xonA *Δ*xseA). **B**) Maintenance of viability of *ΔoriC oriZ^+^* cells in the absence of ExoI, ExoVII or both ExoI and ExoVII. The plate photographs shown are of synthetic lethality assays, as described in Materials and Methods. The relevant genotype of the construct used is shown above each photograph, with the strain number in parentheses. The fraction of white colonies is shown below, with the number of white colonies/total colonies analysed in parentheses. The plasmid used was pAM488 (*xonA^+^*) (see Supplementary Information).

To investigate whether the same is true for cells lacking 3′ exonucleases, we deleted *xonA* and *xseA* in an *oriC^+^ oriZ^+^* background. In contrast to cells lacking RecG, this did not result in an increase in the over-replication in the termination area (Figure 7A). The strong distortion of the replication profile seen in *oriC^+^ oriZ^+^* cells (40,52) is likely to mask the low levels of over-replication seen in *ΔxonA ΔxseA* double mutants (cf. Figures 7A and 2). There appears to be an increase of the marker frequency in the ectopic termination area in *oriC^+^ oriZ^+^ ΔxonA ΔxseA* cells (Figure 7A and Supplementary Figure S2), as reported in cells lacking RecG (16). However, in contrast to the situation in cells lacking RecG, we found that the deletion of *oriC* in *oriC^+^ oriZ^+^ ΔxonA ΔxseA* cells could be achieved without difficulty and the resulting *ΔoriC oriZ^+^ ΔxonA ΔxseA* cells were able to grow without any sign of a growth defect (Figure 7B). The marked difference in the level of over-replication in the absence of either RecG or 3′ exonucleases in cells with an asymmetric replichore arrangement suggest that the molecular intermediates triggering such over-replication in the absence of RecG are likely to differ from the intermediates that arise in the absence of 3′ exonucleases. The *recJ* and *xseA* data clearly suggest a significant degree of overlap in the processing of 3′ flaps, with proteins such as RecG being able to channel some intermediates into different pathways. However, the differences in the genetic requirements shown and the fact that the combination of 3′ exonuclease gene deletions and a deletion of *recG* is synthetically lethal (15) supports the idea that some differences exist. It will require a more detailed analysis to unravel the precise molecular nature of the intermediates involved.

## DISCUSSION

The idea that the 3′ exonucleases ExoI, ExoVII and SbcCD might be involved in termination of DNA replication was originally a prediction derived from our hypothesis that the fusion of two replication forks might lead to the formation of 3′ ssDNA flap structures (15,16). We predicted that the absence of 3′ exonucleases should lead to an accumulation of 3′ flaps, thereby triggering PriA-mediated over-replication of the termination area, which was indeed what we observed when we analysed replication profiles of strains lacking all three 3′ exonucleases (16).

So are 3′ exonucleases involved in termination of DNA replication or might the over-replication observed in cells lacking 3′ exonucleases come from a different cellular process? The data presented in this study confirm that deletions of genes coding for the exonucleases ExoI, ExoVII and SbcCD, and especially combination of deletions, lead to over-replication in the termination area, as reported (16,17). We further demonstrate that the over-replication observed is sufficiently strong to sustain chromosome duplication in the absence of *oriC* firing, but only if the replication fork trap is inactivated by deletion of *tus* and, to a lesser extent, replication-transcription conflicts are alleviated by the destabilization of transcribing RNA polymerase complexes via an rpo* point mutation (Figure 3), very similar to the observations in cells lacking RecG helicase (10,16). The over-replication in the termination area of *ΔxonA ΔxseA* cells (Figure 2) and the lack of growth of *dnaA(ts) ΔxonA ΔxseA* cells compared to dnaA(ts) Δtus rpo* ΔxonA ΔxseA cells at 42°C (Figure 3) strongly suggest that a significant proportion of DNA synthesis is initiated within the fork trap area. While we cannot rule out that some DNA synthesis is initiated away from the fork fusion point, this does not lead to a visible distortion of the replication profile (Figure 2). In addition, we have shown that the absence of all three exonuclease ExoI, ExoVII and SbcCD triggers very high levels of over-replication within the innermost *ter* sites. Thus, while we cannot exclude other possibilities entirely, our data fit well with the idea that these nucleases have a role at the final stages of genome duplication. In contrast, growth of cells in which over-replication is initiated in a variety of chromosomal locations should be independent of the presence or absence of a functional fork trap. Indeed, cells lacking RNase HI, which initiate origin-independent DNA replication in multiple locations across both replichores, including a peak of over-replication within the termination area (10,47,56), have little problem growing in the absence of *oriC* firing even if the replication fork trap is active (10,57).

One particular feature of the termination area in *E. coli* is the replication fork trap formed by *ter*/Tus complexes. Forks stalled at *ter*/Tus complexes might initiate recombination events which might trigger the over-replication observed, as suggested for cells lacking RecG (58). However, growth of *dnaA(ts) ΔxonA ΔxseA* cells depends specifically on the absence of Tus, not its presence (Figure 1), as observed in cells lacking RecG (13,16). In addition, and in contrast to the situation in cells lacking RecG (13), *oriC^+^ oriZ^+^* cells lacking 3′ exonucleases, in which forks are arrested at *ter*/Tus complexes for considerably longer periods, do not show increased levels of over-replication of the termination area. Taken together, our results suggest that a role of *ter*/Tus complexes in triggering the over-replication observed in 3′ exonuclease-deficient cells is unlikely.

The fusion of replication fork complexes is a process that specifically occurs in the termination area. The fact that linearization of the chromosome completely abolishes the over-replication in cells lacking 3′ exonucleases demonstrates that a circular chromosome is required to trigger the over-replication observed. Integration of the *tos* linearization site alone does not change the level of over-replication, which rules out that the integration of *tos* into a cryptic origin is responsible. While we cannot categorically rule out other explanations, one of the most prominent changes caused by linearization of the chromosome is the fact that replication forks will not fuse any more, but will instead reach a chromosome end. Thus, the observations reported are perfectly in line with the hypothesis that the fusion of replication fork complexes can lead to the generation of structures which, if not processed by proteins such as RecG and 3′ exonucleases, will be processed by PriA, thereby leading to over-replication in the termination area.

We were surprised to find that the marker frequency in the termination area of *ΔxonA ΔxseA* cells is only mildly increased (Figure 2) in comparison to our previous results in cells lacking RecG (10,13,16). Could it be that over-replication only occurs in a limited subset of cells? It was shown recently that the loss of sequences corresponding to the terminus area in the replication profile of a *recB* mutant strain came from only a subset of cells (59). However, this idea does not fit well with the fact that a large fraction (just over 60%) of dnaA(ts) Δtus rpo* ΔxonA ΔxseA cells is capable of growing at restrictive temperature (Figure 1). In order to not only initiate origin-independent synthesis but to also sustain growth strong enough to allow colony formation, origin-independent synthesis will have to be initiated in a large fraction of cells, as recently demonstrated in cells lacking RecG helicase (13). What else might influence peak height of over-replication in the termination area? The peak height reported for *ΔxonA ΔsbcCD* cells by Wendel and colleagues (17) is higher than the peak height observed in our *ΔxonA ΔsbcCD* cells (Figure 2). This difference might be due to the use of different strain backgrounds. Wendel *et al.* have used a W3110 derivative (17), which carries a chromosomal inversion that puts the origin in an asymmetric position in relation to the replication fork trap (60). This certainly explains the dramatic levels of over-replication observed in cells lacking RecG (17), as we have demonstrated before that an asymmetric replichore arrangement triggers excessive levels of over-replication in the termination area of *ΔrecG* cells (13,16). In our own MG1655 background, the termination area is opposite *oriC*, with only a very mild asymmetry (40). The replichore asymmetry in W3110 could also explain the differences observed in *ΔxonA ΔsbcCD* cells. The distortion in the termination area caused by the asymmetric replication profile of double origin strains is likely to mask the relatively small levels of over-replication observed in *ΔxonA ΔsbcCD* cells (cf. Figures 7 and 2), in contrast to a construct replicated from a single asymmetric replication origin (17). Thus, it is possible that an asymmetric replichore arrangement indeed leads to a mild increase in the over-replication triggered in the termination area of *ΔxonA ΔxseA* and *ΔxonA ΔsbcCD* cells. However, such an asymmetric replichore arrangement certainly does not exacerbate over-replication as much as reported in *ΔrecG* cells (13,16,17), highlighting that there must be some differences in the molecular substrates that RecG helicase and 3′ exonuclease process.

But why do the levels of over-replication not correlate directly with the ability of cells to grow in the absence of origin firing? One explanation could be the processivity of the over-replication triggered in the termination area. If in the absence of RecG forks are generated which, for some reason, are not processive enough for chromosome duplication, these will contribute towards the over-replication of the termination area, but not towards successful growth of dnaA(ts) Δtus rpo* ΔrecG cells at restrictive temperature. If, in contrast, the majority of replication forks generated in the absence of ExoI and ExoVII are processive enough for genome duplication, a smaller peak of over-replication would still be sufficient to allow for the observed growth of dnaA(ts) Δtus rpo* ΔxonA ΔxseA cells at restrictive temperature. This would allow for the differences in peak heights observed, although we do not have data that directly support this idea and other explanations might apply.

But what are the molecular intermediates that are processed by these nucleases? Wendel and colleagues have suggested that replication forks might move past each other, thereby generating structures that are processed by 3′ exonucleases and RecBCD, explaining why the termination area is degraded in *ΔrecB* cells and over-replicated in *ΔxonA ΔsbcCD* cells (17,18). However, we have recently demonstrated that RecBCD-dependent degradation in the vicinity of the fork fusion point also occurs in strains with a linearized chromosome (61). In cells with a linearized chromosome replication forks run into a chromosome end and certainly cannot move past each other.

While RecG helicase can process a variety of substrates *in vitro*, including 3′ flaps (19–22), exonucleases such as ExoI have a much more defined substrate specificity. Together with the observation that growth of dnaA(ts) Δtus rpo* ΔxonA cells at 42°C is completely suppressed by the *srgA1* point mutation, which renders PriA incapable of unwinding 3′ flap structures, these data strongly suggest that 3′ flap structures accumulate in the absence of ExoI (Figure 5C). The fact that over-replication is suppressed by the *srgA1* allele both in cells lacking ExoI and RecG suggests that 3′ flap structures are key intermediates that can arise as a result of fork fusion events.

While 3′ ssDNA flap structures can be processed by both RecG and 3′ exonucleases (22,26), the mode of processing differs. While 3′ exonucleases such as ExoI will eliminate 3′ flaps by degradation, RecG helicase will convert 3′ into 5′ flaps, as it possesses the necessary activity to unwind the 5′ end at the branch point of a 3′ flap while simultaneously reannealing the 3′ single-strand flap (20,23–25). Our observation that the deletion of *recJ* increases over-replication in *ΔxonA* cells (Figure 6) and that growth of dnaA(ts) Δtus rpo* ΔxonA ΔxseA cells is not fully suppressed by a *srgA1* point mutation, much in contrast to the situation in dnaA(ts) Δtus rpo* ΔxonA cells (Figure 5), strongly supports the idea that *in vivo* 3′ flaps are converted into 5′ flaps and subsequently degraded by 5′ exonucleases, or, in their absence, used by PriA to establish new replication forks (25,51). While we have not yet directly demonstrated the involvement of RecG in this conversion, the fact that *ΔxonA ΔxseA ΔsbcCD* cells require RecG for viability (15) strongly supports the idea that RecG is indeed involved.

**Figure 8.**
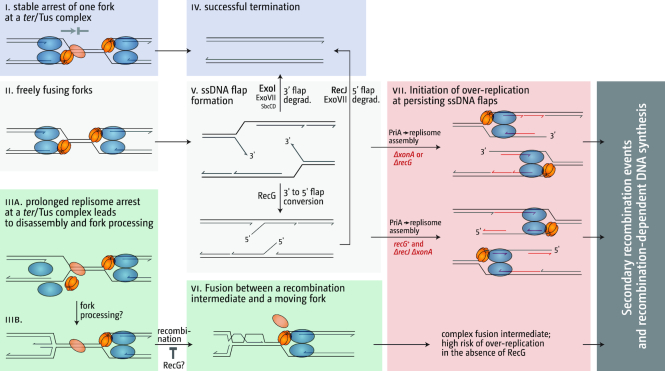
Events associated with the fusion of replication forks in the termination area of *E. coli*. Different fork fusion conditions are shown on the left (panels i, ii and iii), leading to different fork fusion intermediates (panels iv, v and vi), which have to be processed to prevent PriA-dependent over-replication (panel vii) and lead to successful termination. See text for further details.

Thus, while not categorically ruling out other explanations, the data presented here strongly support the idea that 3′ flaps arise as a result of replication fork fusion events and are either degraded by 3′ exonucleases or converted by RecG into 5′ flaps and degraded subsequently by 5′ exonucleases. As for both dnaA(ts) ΔrecG Δtus rpo* and dnaA(ts) ΔxonA ΔxseA Δtus rpo* cells about 60% of cells are able to form colonies at 42°C, a certain fraction of the fork fusion events do not appear to result in aberrant fusion intermediates, resulting in a successful termination event (Figure 8 iv). However, if fusion intermediates such as 3′ flaps are formed in the absence of either RecG or 3′ exonucleases (Figure 8v), PriA will have a chance to load new replication forks, thereby causing the over-replication observed (Figure 8vii).

In addition to the observed similarities in cells lacking RecG and 3′ exonucleases, our data also highlight that distinct differences exist between both pathways. Cells lacking RecG show robust levels of over-replication despite the presence of 3′ exonucleases. While this might be caused simply by an over-saturation of the system with 3′ flap intermediates, the particularly low levels of over-replication in the termination area in *ΔxonA* and *ΔxonA ΔxseA* cells could also be in line with the idea that there might be a difference in at least some of the intermediates that accumulate or, alternatively, in the processivity in the forks established, as already discussed above. This idea is additionally supported by our observation that introduction of a second, ectopic origin has, if any, only a moderate effect in cells lacking 3′ exonucleases, while extreme levels of over-replication are triggered in the termination area if RecG is missing (13,16). We have suggested before that in cells lacking RecG the difference is triggered by the disassembly of replication forks (13). We have postulated that a fusion event between a fork stably arrested at a *ter*/Tus complex and a moving fork has little chance of generating a 3′ flap intermediate, as both Tus protein and DnaB helicase act as a ‘buffer’ for the opposing fork (Figure 8ii). In contrast, two freely fusing forks and especially a situation where one fork is partially disassembled while the other is freely moving increases the danger of the displacement of the nascent leading strand (Figure 8i and iii). If this was the case then the fact that the prolonged arrest of forks at *ter*/Tus does not make any difference to the over-replication in cells lacking 3′ exonucleases suggests that 3′ exonucleases do not act in this particular situation. It was shown before that exonucleases can gain access to blocked replication forks, especially in the absence of the RecBCD (18,59,61–63). However, our recent analysis highlights that in this situation SbcCD has a particularly prominent role (61). In contrast, for the over-replication observed in this study it appears that ExoI is by far the most prominent nuclease, whereas the effect of SbcCD is not particularly pronounced (Figure 1). We have proposed before that the effect of RecG on the over-replication of the termination area is two-fold. While RecG will process 3′ flap structures (Figure 8v), RecG will also be involved in preventing successful recombination events that take place as a consequence of the over-replication triggered at 3′ flaps (16). Indeed, Azeroglu and colleagues recently showed that repair of dsDNA breaks in the absence of RecG leads to divergent DNA replication at the location of the dsDNA break (58). If RecG is involved both in the elimination of 3′ flaps and also in preventing over-replication triggered at recombination events arising as a result of the over-replication in the termination area (Figure 8vi), this might well explain the differences observed in cells lacking RecG and 3′ exonucleases. It will require a more detailed analysis to identify the precise molecular nature of the intermediates arising and how these are processed to normally prevent over-replication in the termination area to get a clear picture of the events associated with the fusion of two replication forks.

## DATA AVAILABILITY

All relevant raw sequencing data can be accessed at the European Nucleotide Archive (http://www.ebi.ac.uk/ena/data/view/PRJEB28600).

## Supplementary Material

Supplementary Data
