# Supplementary material for: A role for 3′ exonucleases at the final stages of chromosome duplication in Escherichia coli
Source: Nucleic Acids Res. 2018 Dec 14;47(4):1847–60. doi: 10.1093/nar/gky1253 (PMC6393302; doi:10.1093/nar/gky1253)
Supplement: Supplementary Data [file gky1253_supplemental_files.pdf]

Supplementary Information

A role for 3′ and 5′ exonucleases at the final stages of chromosome duplication in *Escherichia coli*

Sarah L. Midgley-Smith, Juachi U. Dimude and Christian J. Rudolph*

*Corresponding author: christian.rudolph@brunel.ac.uk

Division of Biosciences, College of Health and Life Sciences,
Brunel University London, Uxbridge, UB8 3PH, UK

SUPPLEMENTARY METHODS

Marker frequency analysis by deep sequencing

Marker frequency analysis by Deep Sequencing was performed as described previously (1–3) with only minor modifications. Samples from cultures of a strain grown over night in LB broth were diluted 100-fold in fresh LB broth and incubated with vigorous aeration until an A_{600} reached 0.48 at 37°C to ensure they were in exponential growth conditions. Cultures were then diluted a second time 100-fold in pre-warmed fresh broth and grown again until an A_{600} of 0.48 was reached. Samples from these exponential phase cultures were flash-frozen in liquid nitrogen at this point for subsequent DNA extraction. For wild type, incubation of the remaining culture was continued until several hours after the culture had saturated and showed no further increase in the A_{600}. A further sample (stationary phase) was frozen at this point. DNA was then extracted using the GenElute Bacterial Genomic DNA Kit (Sigma-Aldrich). Marker frequency analysis was performed using Illumina HiSeq 2500 sequencing (fast run) to measure sequence copy number. FastQC was used for a basic metric of quality control in the raw data. Bowtie2 was used to align the sequence reads to the reference. Samtools was used to calculate the enrichment of uniquely mapped sequence tags in 1 kb windows.

For presentation of the data as a marker frequency replication profile the raw read counts for each construct were divided by the average of all read counts across the entire genome to correct for the somewhat different absolute numbers of aligned reads in the various samples. The normalised read count values for each exponentially growing sample were then divided by the corresponding normalised read count value from a stationary (non-replicating) sample. This division “cleans” the raw data significantly, because data points which are outliers caused by technical aspects (precise sequence environment interfering with library preparation or similar issues) will be similarly distorted both in the exponential and the stationary samples.

Bacteriophage N15 infection and lysogen preparation

For preparation of a phage N15 plate lysate, cells from an overnight culture grown in Mu were spun down and resuspended in 10 mM $MgSO_4$. Phage N15 was diluted in M9 minimal medium without glucose, 10^5–10^6 phage particles mixed with 100 µl of the prepared cells and the mixture incubated 5 min at room temperature. 2.5 ml Mu were added, followed by 2.5 ml molten Mu top agar (45°C), mixed and poured on top of a fresh Mu plate. Plates were incubated upright at 37°C for 7 h. 2 ml of M9 minimal medium without glucose were pipetted onto the plate and the top agar overlay was scraped off and transferred into a centrifugation tube. 0.5 ml chloroform was added and cell debris and top agar removed by centrifugation (10,000 rpm, 4°C). For determination of the phage N15 titre as well as infection of target strains, N15 was diluted in M9 minimal medium without glucose. 100 µl of the target or tester

strain was mixed with 2.5 ml molten Mu top agar (45°C) and poured on top of a fresh Mu plate. 10 µl drops of appropriate dilutions of a lysate were placed on the top agar and incubated at room temperature until dry. The plate was then inverted and incubated over night at 37°C. For infection and lysogen preparations, 10 µl drops containing ~10^4 phage particles were used.

Pulsed-field gel electrophoresis for confirmation of chromosome linearization

For verification of chromosome linearization high molecular weight chromosomal DNA digested with the rare cutter *Not*I was separated via pulsed-field gel electrophoresis (PFGE). The *tos* site is located in the 273.6 kb *Not*I fragment between positions 1,337,601 and 1,611,219 (Figure S1C) and cleavage by TelN splits it into two fragments, one of which is 251.2 kb and the other one 22.4 kb (Figure S1E and S1F). The presence of these extra band can be easily verified by PFGE. Cells of an exponentially growing culture were resuspended in 85 µl TEE buffer (10 mM Tris • HCl, 10 mM EGTA, 100 mM EDTA, pH 8.0), containing 0.05% lauroylsarcosine and 0.5% SDS. 85 µl of liquid 1.4% low melting point agarose was added and the mixture solidified in a disposable plug former (Bio-Rad) at 4°C. Plugs were treated with 10 mg/ml lysozyme in 3 ml TEE buffer containing 0.05% lauroylsarcosine and 0.5% SDS for 2 h at 37°C and then at 52°C overnight with 5 mg/ml proteinase K in 3 ml TEE containing 1% SDS. Plugs were washed in TEE for 30 min at 37°C, treated with 1 mM phenylmethane sulphonyl fluoride (freshly prepared as 100 mM stock solution in methanol) in fresh TEE for 1 h at 37°C, washed in fresh TEE for 30 min at 37°C and finally in 0.1 × TEE for 30 min at 37°C. The plugs were subsequently transferred into 300 µl restriction enzyme buffer and incubated for 30 min at room temperature, the buffer changed and 25 u of *Not*I (NEB) added. Chromosomal DNA was digested overnight and the fragments separated on a 0.8% agarose gel (Bio-Rad pulse field certified agarose) in 0.5 × TBE using a CHEF Mapper PFGE system (Bio-Rad), running with a gradient voltage of 6 V/cm, an included angle of 120°, and initial and final switch times of 1.65 and 32.45 sec, respectively, with a run time of 20 h at 14°C.

Plasmids used in this study

Plasmid pAM488 (*xonA*$^+$) was generated by cloning the multiple cloning site (MCS) of pGEM into the *Apa*I site of pRC7. This allowed cloning of the entire *xonA* gene, including its promoter region, into the *Bam*HI and *Hin*dIII sites of the integrated MCS.

SUPPLEMENTARY TABLE

Table S1: *Escherichia coli* K-12 strains

Strain number	Relevant Genotype[a]	Construction or source[c]
**General P1 donors**		
DL729	*ΔsbcCD::kan recD1009 supE supF*	David Leach
RUC663	*tnaA::*Tn10 *dnaA46*	Tove Atlung
STL2694	*xonAΔ300::cat thr-1 leuB6 proA2 supE44 kdg51 rfbD1 araC14 lacY1 galK2 xyl-5 mtl-1tsx-33 rpsL31 rac*$^{-}$	Susan Lovett
**MG1655 derivatives**		
MG1655	F^{-} *rph-1*	(4)
AM1775	*Δtus::cat*	(5)
AM1874	*ΔxseA::dhfr*	(6)
APS345	*att*Tn7*::lacO240-kan zdd/e::tetO240-gen*	(7)
AS1103	*ΔlacIZYA ΔsbcCD::spc ΔxseA::dhfr ΔxonA::apra* pAM401 pAST116	N7684 × pAST116 to Km^{r}
AU1054	*dnaA46 tnaA::*Tn10	(7)
JD1104	*ΔlacIZYA srgA1 argE86::*Tn10	JJ1264 × P1.RCe300 to Tc^{r}
JD1107	*ΔlacIZYA srgA1 rpoB*35*	JD1104 × P1.RCe395 to Tc^{s} Arg^{+}
JD1152	*priA300 rpoB*35 dnaA46 tnaA::*Tn10	N5535 × P1.RUC663 to Tc^{r}
JD1153	*ΔlacIZYA srgA1 rpoB*35 dnaA46 tnaA::*Tn10	JD1107 × P1.RUC663 to Tc^{r}
JD1257	*ΔlacIZYA argE86::*Tn10	TB28 × P1.N4837 to Tc^{r}
JD1350	*ΔlacIZYA oriZ-<cat> ΔxonA::apra ΔoriC::kan* pAM488	SLM1210 × P1.RCe576 to Km^{r}
JD1351	*ΔlacIZYA oriZ-<cat> ΔxseA::dhfr ΔxonA::apra ΔoriC::kan* pAM488	SLM1215 × P1.RCe576 to Km^{r}
JJ1261	*ΔlacIZYA metB1*	JJ1257 × P1.N4441 Arg^{+} Met^{-}
JJ1264	*ΔlacIZYA srgA1*	JJ1261 × P1.N3695 to Met^{+}
N3695	*ΔrecG263::kan srgA1*	(8)
N4441	*ΔrecG263::kan metB1*	A.A. Al-Deib and R.G. Lloyd, unpublished
N4837	*argE::*Tn10	(9)
N4849	*rpoB*35*	(9)
N4934	*recJ284::*Tn10	(10)
N5286	*xonAΔ300::cat*	MG1655 × P1.STL2694 to Cm^{r}
N5296	*xonAΔ300::cat ΔsbcCD::kan*	N5286 × P1.DL729 to Km^{r}
N5535	*priA300 rpoB*35*	(9)
N7684	*ΔlacIZYA ΔsbcCD::spc ΔxseA::dhfr ΔxonA::apra* pAM401	(6)
RCe203	*tnaA::*Tn10 *dnaA46 Δtus::kan*	(5)
RCe267	*rpoB*35 Δtus::cat dnaA46 tnaA::*Tn10	(5)
RCe300	*att*Tn7*::lacO240-kan zdd/e::tetO240-gen argE::*Tn10	APS345 × P1.N4837 to Tc^{r} Arg^{-}

RCe395	rpoB*35 dnaA46 tnaA::Tn10 ΔrnhA::cat tus1::dhfr ΔoriC::kan	(5)
RCe427	tos-kan	(5)
RCe504	oriZ-<cat>	(1)
RCe528	rpoB*35 Δtus::cat dnaA46 tnaA::Tn10 ΔxonA::apra	RCe267 × P1.AS1103 to Apra^{r}
RCe544	ΔlacIZYA oriZ-<cat>	(1)
RCe553	rpoB*35 Δtus::cat dnaA46 tnaA::Tn10 ΔsbcCD::kan	RCe267 × P1.N5296 to Km^{r}
RCe554	rpoB*35 Δtus::cat dnaA46 tnaA::Tn10 ΔsbcCD::kan ΔxonA::apra	RCe553 × P1.AS1103 to Apra^{r}
RCe562	ΔsbcCD::kan	MG1655 × P1.N5296 to Km^{r}
RCe563	ΔxonA::apra	MG1655 × P1.AS1103 to Apra^{r}
RCe569	ΔxonA::apra ΔsbcCD::kan	RCe563 × P1.N5296 to Km^{r}
RCe576	rpoB*35 oriZ-<cat> tus1::dhfr ΔoriC::kan[b]	(1)
SLM1107	priA300 rpoB*35 dnaA46 tnaA::Tn10 Δtus::cat	JD1152 × P1.AM1775 to Cm^{r}
SLM1108	ΔlacIZYA srgA1 rpoB*35 dnaA46 tnaA::Tn10 Δtus::cat	JD1153 × P1.AM1775 to Cm^{r}
SLM1109	priA300 rpoB*35 dnaA46 tnaA::Tn10 Δtus::cat ΔxonA::apra	SLM1107 × P1.AS1103 to Apra^{r}
SLM1110	ΔlacIZYA srgA1 rpoB*35 dnaA46 tnaA::Tn10 Δtus::cat ΔxonA::apra	SLM1108 × P1.AS1103 to Apra^{r}
SLM1170	tnaA::cat	This study
SLM1171	ΔlacIZYA srgA1 pAM488	JJ1264 × pAM488 to Ap^{r}
SLM1172	ΔlacIZYA srgA1 ΔxonA::apra	SLM1171 × P1.RCe563 to Ap^{s} Apra^{r}
SLM1174	tos-kan ΔxonA::apra	RCe427 × P1.RCe563 to Apra^{r}
SLM1178	ΔxonA::apra recJ284::Tn10	RCe563 × P1.N4934 to Tc^{r}
SLM1184	rpoB*35 ΔxseA::dhfr	N4849 × P1.AM1874 to Tm^{r}
SLM1185	ΔxseA::dhfr	MG1655 × P1.AM1874 to Tm^{r}
SLM1186	ΔlacIZYA srgA1 ΔxonA::apra ΔxseA::dhfr	SLM1172 × P1.AM1874 to Tm^{r}
SLM1187	tos-kan ΔxonA::apra ΔxseA::dhfr	SLM1174 × P1.AM1874 to Tm^{r}
SLM1188	ΔxonA::apra recJ284::Tn10 ΔxseA::dhfr	SLM1178 × P1.AM1874 to Tm^{r}
SLM1189	dnaA46 tnaA::cat	AU1054 × P1.SLM1170 to Cm^{r}
SLM1190	rpoB*35 ΔxseA::dhfr Δtus::kan	SLM1184 × P1.RCe203 to Km^{r}
SLM1191	rpoB*35 ΔxseA::dhfr Δtus::kan ΔxonA::apra	SLM1190 × P1.RCe563 to Apra^{r}
SLM1194	rpoB*35 ΔxseA::dhfr Δtus::kan ΔxonA::apra dnaA46 tnaA::Tn10	SLM1191 × P1.RUC663 to Tc^{r}
SLM1195	rpoB*35 Δtus::kan	N4849 × P1.RCe203 to Km^{r}
SLM1196	rpoB*35 ΔxseA::dhfr Δtus::cat	SLM1184 × P1.AM1775 to Cm^{r}
SLM1198	priA300 rpoB*35 dnaA46 tnaA::Tn10 Δtus::cat ΔxonA::apra ΔxseA::dhfr	SLM1109 × P.1AM1874 to Tm^{r}
SLM1199	ΔlacIZYA srgA1 rpoB*35 dnaA46 tnaA::Tn10 Δtus::cat ΔxonA::apra ΔxseA::dhfr	SLM1110 × P1AM1875 to Tm^{r}
SLM1201	rpoB*35 Δtus::kan ΔxonA::apra	SLM1195 × P1.RCe563 to Apra^{r}
SLM1202	rpoB*35 ΔxseA::dhfr Δtus::cat ΔxonA::apra	SLM1196 × P1.RCe563 to Apra^{r}
SLM1203	ΔxseA::dhfr ΔxonA::apra	SLM1185 × P1.RCe563 to Apra^{r}

SLM1204	*ΔxseA::dhfr recJ284::*Tn*10*	SLM1185 × P1.N4934 to Tc^r
SLM1206	*ΔlacIZYA oriZ-<cat> ΔxonA::apra*	RCe544 × P1.RCe563 to Apra^r
SLM1208	*ΔlacIZYA oriZ-<cat> ΔxseA::dhfr*	RCe544 × P1.AM1874 to Tm^r
SLM1209	*ΔxseA::dhfr ΔsbcCD::kan*	SLM1185 × P1.RCe562 to Km^r
SLM1210	*ΔlacIZYA oriZ-<cat> ΔxonA::apra* pAM488	SLM1206 × pAM488 to Ap^r
SLM1211	*ΔlacIZYA oriZ-<cat> ΔxseA::dhfr* pAM488	SLM1208 × pAM488 to Ap^r
SLM1212	*tos-kan ΔxonA::apra ΔxseA::dhfr* N15 lysogen	SLM1187 × N15 to N15^r
SLM1213	*ΔxseA::dhfr ΔxonA::apra* N15 lysogen	SLM1203 × N15 to N15^r
SLM1215	*ΔlacIZYA oriZ-<cat> ΔxseA::dhfr ΔxonA::apra* pAM488	SLM1211 × P1.RCe563 to Apra^r Ap^r
SLM1217	*ΔlacIZYA oriZ-<cat> ΔxseA::dhfr ΔxonA::apra*	Plasmid-free derivative of SLM1215
SLM1218	*rpoB*35 Δtus::kan ΔxonA::apra dnaA46 tnaA::cat*	SLM1201 × P1.SLM1189 to Cm^r
SLM1219	*rpoB*35 ΔxseA::dhfr Δtus::cat dnaA46 tnaA::*Tn*10*	SLM1196 × P1.RUC663 to Tc^r
SLM1220	*rpoB*35 ΔxseA::dhfr Δtus::cat ΔxonA::apra tos-kan*	SLM1202 × P1.RCe427 to Km^r
SLM1221	*rpoB*35 ΔxseA::dhfr Δtus::cat ΔxonA::apra dnaA46 tnaA::*Tn*10*	SLM1202 × P1.RUC663 to Tc^r
SLM1222	*rpoB*35 Δtus::kan dnaA46 tnaA::cat*	SLM1195 × P1.SLM1189 to Cm^r
SLM1223	*rpoB*35 ΔxseA::dhfr Δtus::cat dnaA46 tnaA::*Tn*10 ΔsbcCD::kan*	SLM1219 × P1.RCe562 to Km^r
SLM1224	*rpoB*35 Δtus::kan ΔxonA::apra dnaA46 tnaA::cat recJ284::*Tn*10*	SLM1218 × P1.RCe4934 to Tc^r
SLM1225	*rpoB*35 ΔxseA::dhfr Δtus::cat ΔxonA::apra tos-kan dnaA46 tnaA::*Tn*10*	SLM1220 × P1.RUC663 to Tc^r
SLM1226	*rpoB*35 ΔxseA::dhfr Δtus::cat dnaA46 tnaA::*Tn*10 ΔsbcCD::kan ΔxonA::apra*	SLM1223 × P1.RCe563 to Apra^r
SLM1230	*rpoB*35 ΔxseA::dhfr Δtus::cat ΔxonA::apra tos-kan dnaA46 tnaA::*Tn*10* N15 lysogen	SLM1225 × N15 to N15^r
SLM1232	*rpoB*35 ΔxseA::dhfr Δtus::cat ΔxonA::apra dnaA46 tnaA::*Tn*10* N15 lysogen	SLM1221 × N15 to N15^r
SLM1233	*rpoB*35 Δtus::kan dnaA46 tnaA::cat recJ284::*Tn*10*	SLM1222 × P1.N4934 to Tc^r
SLM1236	*rpoB*35 ΔxonA::apra*	N4849 × P1.RCe563 to Apra^r
SLM1238	*rpoB*35 ΔxonA::apra ΔxseA::dhfr*	SLM1236 × P1.AM1874 to Tm^r
SLM1242	*ΔxseA::dhfr ΔxonA::apra Δtus::cat*	SLM1203 × P1.AM1775 to Cm^r
SLM1244	*ΔxseA::dhfr ΔxonA::apra Δtus::cat dnaA46 tnaA::*Tn*10*	SLM1242 × P1.RUC663 to Tc^r
SLM1245	*rpoB*35 ΔxonA::apra ΔxseA::dhfr dnaA46 tnaA::*Tn*10*	SLM1238 × P1.RUC663 to Tc^r
SLM1246	*ΔxseA::dhfr ΔxonA::apra dnaA46 tnaA::*Tn*10*	SLM1203 × P1.RUC663 to Tc^r
TB28	*ΔlacIZYA*	(11)

a – Only the relevant additional genotype of the derivatives is shown. The abbreviations *apra*, *kan*, *cat* and *dhfr* refer to insertions conferring resistance to apramycin (Apra^r), kanamycin (Km^r), chloramphenicol (Cm^r) and trimethoprim (Tm^r), respectively. Tn*10* indicates the presence of a transposon 10 integration, which confers resistance to tetracycline (Tc^r). '<>' indicates the use of *frt* sites, where *frt* stands for the 34 bp recognition site of the FLP/*frt* site-directed

recombination system. Thus, <*cat*> refers to a kanamycin marker flanked by an *frt* site either side. *tos* refers to the telomerase occupancy site from the bacteriophage N15 genome followed by a kanamycin resistance cassette (12).

b – *ΔoriC* refers to a replacement of the entire origin region (754 bp) including DnaA boxes and 13mers as well as the entire *mioC* gene by a kanamycin resistance cassette (5).

c – The term "× N15 to N15^r" refers to isolation of *E. coli* cells lysogenized with bacteriophage N15. These cells can be identified by their resistance to re-infection with N15 (see Supplementary Methods). Plasmids relevant to this study are described in Supplementary Methods.

Table S2: Growth and DNA damage characteristics of *dnaA(ts) Δtus rpo** cells lacking 3' exonucleases

Strain No.	Relevant Genotype	Doubling Time [min]	Colony forming units at A_{600} = 0.35	UV sensitivity (60 J/m²)	MMC sensitivity (0.5 µg/ml)	UV + MMC sensitivity (30 J/m² + 0.5 µg/ml)
RCe267	*dnaA(ts) Δtus rpo**	41.7	1.3×10^8	(+)[a]	(+)[a]	(+)[a]
RCe528	*dnaA(ts) Δtus rpo*ΔxonA*	51.9	1.5×10^8	(+)	(+)	(+)
SLM1219	*dnaA(ts) Δtus rpo* ΔxseA*	51.4	1.2×10^8	(+)	(+)	(+)
RCe553	*dnaA(ts) Δtus rpo* ΔsbcCD*	50.2	1.7×10^8	(+)	(+)	(+)
SLM1194	*dnaA(ts) Δtus rpo* ΔxonA ΔxseA*	52.0	1.6×10^8	(+)	(+)	(+)
RCe554	*dnaA(ts) Δtus rpo* ΔxonA ΔsbcCD*	52.1	1.4×10^8	(+)	(+)	(+)
SLM1223	*dnaA(ts) Δtus rpo* ΔxseA ΔsbcCD*	53.2	1.1×10^8	(+)	(+)	(+)
SLM1226	*dnaA(ts) Δtus rpo* ΔxonA ΔxseA ΔsbcCD*	55.6	1.2×10^8	+++	+	+++
SLM1110	*dnaA(ts) Δtus rpo*ΔxonA srgA1*	47.4	1.2×10^8	(+)	(+)	(+)
SLM1199	*dnaA(ts) Δtus rpo* ΔxonA ΔxseA srgA1*	47.6	1.8×10^8	(+)	(+)	(+)
SLM1198	*dnaA(ts) Δtus rpo* ΔxonA ΔxseA priA300*	50.8	1.1×10^8	(+)	(+)	(+)
SLM1232	*dnaA(ts) Δtus rpo* ΔxonA ΔxseA* N15 lysogen	56.6	1.6×10^8	(+)	(+)	(+)
SLM1225	*dnaA(ts) Δtus rpo* ΔxonA ΔxseA tos*	51.5	1.5×10^8	(+)	(+)	(+)
SLM1230	*dnaA(ts) Δtus rpo* ΔxonA ΔxseA tos* N15 lysogen	54.3	1.2×10^8	(+)	(+)	(+)
SLM1244	*dnaA(ts) Δtus ΔxonA ΔxseA*	55.6	1.0×10^8	++	+	+++
SLM1245	*dnaA(ts) rpo* ΔxonA ΔxseA*	47.6	1.4×10^8	+	(+)	(+)
SLM1246	*dnaA(ts) ΔxonA ΔxseA*	54.4	0.8×10^8	++	++	+++
SLM1233	*dnaA(ts) Δtus rpo* ΔrecJ*	48.0	1.2×10^8	++	(+)	+
SLM1224	*dnaA(ts) Δtus rpo*ΔxonA ΔrecJ*	55.6	1.1×10^8	++	+	+++

a – The *dnaA46* allele confers a very mild sensitivity to DNA damaging agents, such as UV and mitomycin C (MMC), in comparison to MG1655 wild type cells, which we have indicated here by a (+). All constructs where measured against the sensitivity of the *dnaA46 Δtus rpo** control. Only cells lacking all three 3' exonucleases show a significantly increased sensitivity to DNA damage, as reported before (6). Similarly, the synergistic interaction of *recJ* with deletions of genes encoding for 3' exonucleases was observed before (6).

SUPPLEMENTARY FIGURE LEGENDS

Suppl. Figure 1. Linearisation of the *Escherichia coli* chromosome. **A)** Schematic representation of *tosRL* processing by the bacteriophage N15 telomerase TelN. **B)** Schematic representation of the area around the *dif* dimer resolution site, with and without integrated *tosRL-kan* site. The linearisation verification primers are shown in green (for primer sequences see (12)) and the PCR product sizes in wild type cells und integrants are indicated. **C)** PCR products generated with the linearisation verification primers for wild type cells (lane 1), *ΔxonA ΔxseA tos-kan* cells (lane 2) and a *ΔxonA ΔxseA tos-kan* construct lysogenized with phage N15 (lane 3). The shift of the PCR product size in lane 2 indicates the presence of the *tos-kan* cassette. Linearisation of the chromosome (lanes 3) prevents formation of a PCR product since the chromosome is interrupted between the primer binding sites. The absence of a detectable PCR product confirms that the amount of circular chromosomes unprocessed by TelN in the population is very low, as reported (12). **D–G)** Verification of chromosome linearisation by pulse field gel electrophoresis. If the *tos* site is cleaved by TelN, an additional band becomes visible on PFGE gels. The *tos* site is located in the 273.6 kb *Not*I fragment between positions 1,337,601 and 1,611,219 (C, highlighted in green) and cleavage by TelN splits it into two fragments, one of which is 251.2 kb and the other one 22.4 kb (E and F, highlighted in green). The 251.2 kb fragment moves into the quadruplet around 250 kb and thus is hidden in between other fragments (E). The smaller 22.4 kb fragment, however, becomes visible as an additional fragment at the bottom of the gel highlighted by a black arrow (D and E). A negative image is shown for clarity. The chromosomal DNA was prepared from *ΔxonA ΔxseA* N15 lysogen (SLM1213) in lane 1, *ΔxonA ΔxseA tos-kan* (SLM1187) in lane 2 and *ΔxonA ΔxseA tos-kan* N15 lysogen (SLM1212) in lane 3.

Suppl. Figure 2. DNA replication profiles of *E. coli* cells with two replication origins in the absence of 3' exonucleases ExoI (*ΔxonA*) and ExoVII (*ΔxseA*). Data in panels i–iii are re-plotted from Figure 7. To highlight potential over-replication in the native and ectopic termination areas, an overlay of the profiles of *oriC*$^+$ *oriZ*$^+$ *ΔxonA* (green) and *oriC*$^+$ *oriZ*$^+$ *ΔxseA* (orange) as well as *oriC*$^+$ *oriZ*$^+$ *ΔxonA* (green) and *oriC*$^+$ *oriZ*$^+$ *ΔxonA ΔxseA* (orange) was generated. Profiles were aligned according to their origin peak heights to reflect the similar doubling times, as discussed previously (13). While the profiles of *ΔxonA* and *ΔxseA* derivatives showed now significant differences, some elevation in both termination areas can be seen in *oriC*$^+$ *oriZ*$^+$ *ΔxonA ΔxseA* cells.

REFERENCES

1. Ivanova,D., Taylor,T., Smith,S.L., Dimude,J.U., Upton,A.L., Mehrjouy,M.M., Skovgaard,O., Sherratt,D.J., Retkute,R. and Rudolph,C.J. (2015) Shaping the landscape of the *Escherichia coli* chromosome: replication-transcription encounters in cells with an ectopic replication origin. *Nucleic Acids Res.*, **43**, 7865–7877.

2. Müller,C.A., Hawkins,M., Retkute,R., Malla,S., Wilson,R., Blythe,M.J., Nakato,R., Komata,M., Shirahige,K., de Moura,A.P.S., *et al.* (2014) The dynamics of genome replication using deep sequencing. *Nucleic Acids Res.*, **42**, e3.

3. Skovgaard,O., Bak,M., Løbner-Olesen,A. and Tommerup,N. (2011) Genome-wide detection of chromosomal rearrangements, indels, and mutations in circular chromosomes by short read sequencing. *Genome Res.*, **21**, 1388–1393.

4. Bachmann, B J (1996) Derivations and Genotypes of Some Mutant Derivatives of *Escherichia coli* K-12. In *Escherichia coli and Salmonella Cellular and Molecular Biology*. ASM Press.

5. Rudolph,C.J., Upton,A.L., Stockum,A., Nieduszynski,C.A. and Lloyd,R.G. (2013) Avoiding chromosome pathology when replication forks collide. *Nature*, **500**, 608–611.

6. Rudolph,C.J., Mahdi,A.A., Upton,A.L. and Lloyd,R.G. (2010) RecG protein and single-strand DNA exonucleases avoid cell lethality associated with PriA helicase activity in *Escherichia coli*. *Genetics*, **186**, 473–492.

7. Rudolph,C.J., Upton,A.L. and Lloyd,R.G. (2007) Replication fork stalling and cell cycle arrest in UV-irradiated *Escherichia coli*. *Genes Dev.*, **21**, 668–681.

8. Al-Deib,A.A., Mahdi,A.A. and Lloyd,R.G. (1996) Modulation of recombination and DNA repair by the RecG and PriA helicases of *Escherichia coli* K-12. *J. Bacteriol.*, **178**, 6782–6789.

9. Mahdi,A.A., Buckman,C., Harris,L. and Lloyd,R.G. (2006) Rep and PriA helicase activities prevent RecA from provoking unnecessary recombination during replication fork repair. *Genes Dev.*, **20**, 2135–2147.

10. Rudolph,C.J., Upton,A.L. and Lloyd,R.G. (2008) Maintaining replication fork integrity in UV-irradiated *Escherichia coli* cells. *DNA Repair*, **7**, 1589–1602.

11. Bernhardt,T.G. and de Boer,P.A.J. (2004) Screening for synthetic lethal mutants in *Escherichia coli* and identification of EnvC (YibP) as a periplasmic septal ring factor with murein hydrolase activity. *Mol. Microbiol.*, **52**, 1255–1269.

12. Cui,T., Moro-oka,N., Ohsumi,K., Kodama,K., Ohshima,T., Ogasawara,N., Mori,H., Wanner,B., Niki,H. and Horiuchi,T. (2007) *Escherichia coli* with a linear genome. *EMBO Rep.*, **8**, 181–187.

13. Midgley-Smith,S.L., Dimude,J.U., Taylor,T., Forrester,N.M., Upton,A.L., Lloyd,R.G. and Rudolph,C.J. (2018) Chromosomal over-replication in *Escherichia coli recG* cells is triggered by replication fork fusion and amplified if replichore symmetry is disturbed. *Nucleic Acids Res.*, **46**, 7701–7715.

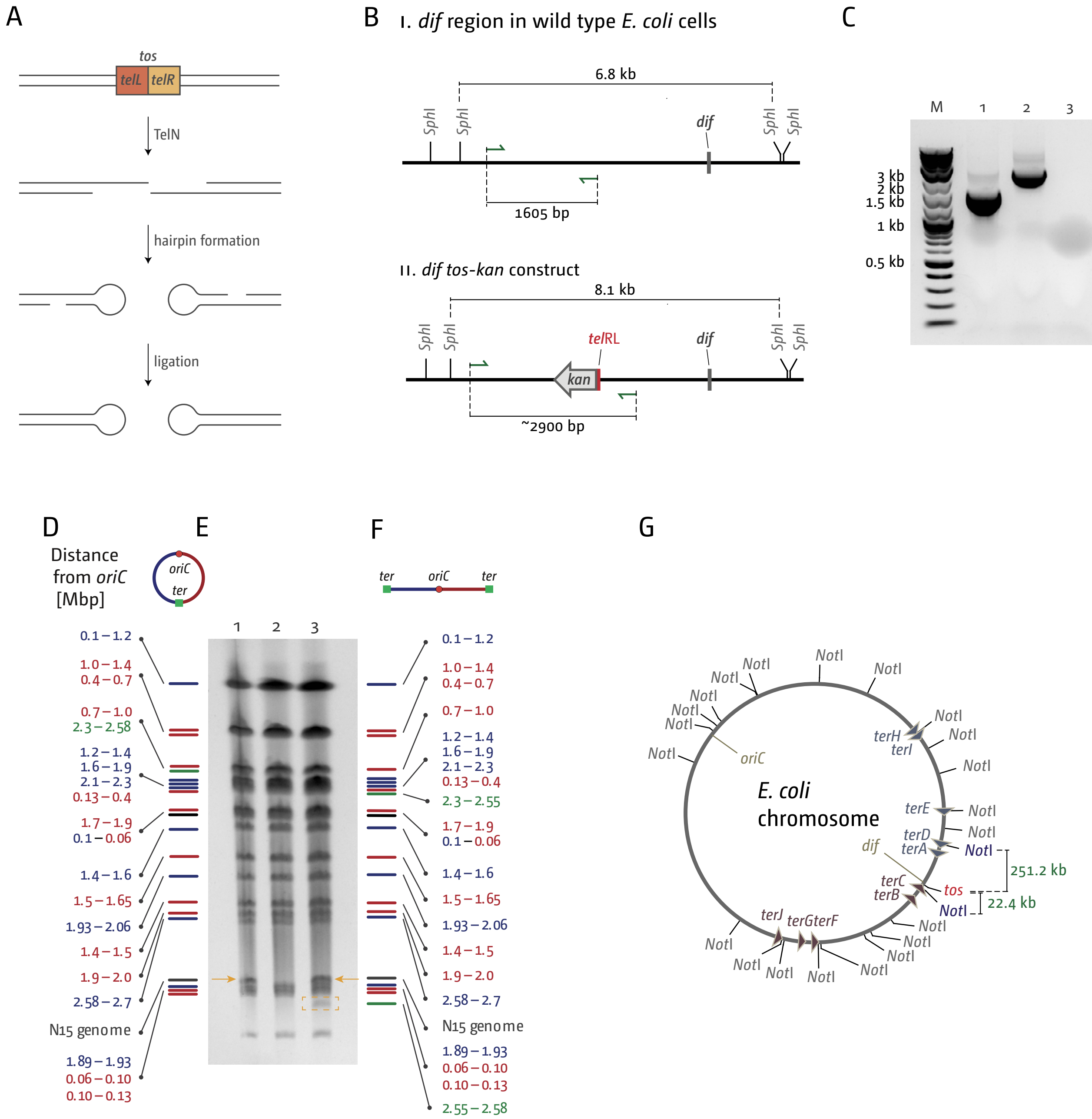

Midgley-Smith *et al.* Figure S1

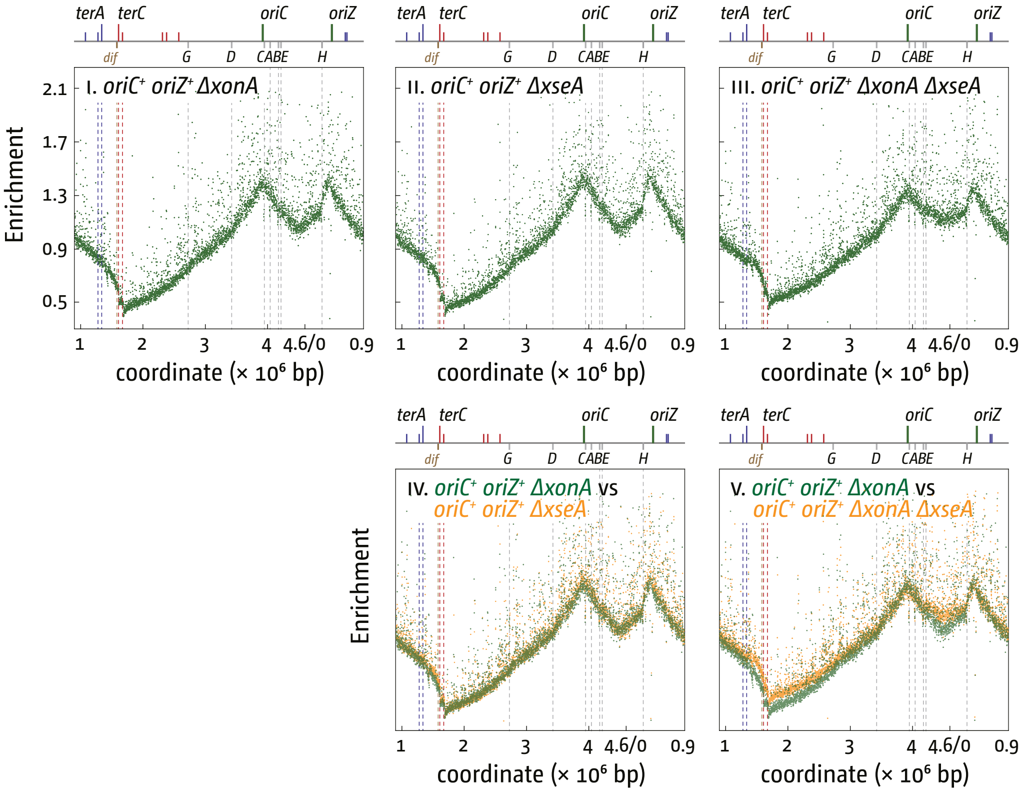

Midgley-Smith *et al.* Figure S2
